# Impact of left ventricular outflow tract ellipticity on the grading of aortic stenosis in patients with normal ejection fraction

**DOI:** 10.1186/s12968-017-0344-8

**Published:** 2017-03-15

**Authors:** Frédéric Maes, Sophie Pierard, Christophe de Meester, Jamila Boulif, Mihaela Amzulescu, David Vancraeynest, Anne-Catherine Pouleur, Agnès Pasquet, Bernhard Gerber, Jean-Louis Vanoverschelde

**Affiliations:** 1 Pôle de Recherche Cardiovasculaire, Institut de Recherche Expérimentale et Clinique, Université Catholique de Louvain, Brussels, Belgium; 2 Division of Cardiology, Cliniques Universitaires Saint-Luc, Brussels, Belgium

**Keywords:** Aortic stenosis, Gradient, Left ventricular outflow tract

## Abstract

**Background:**

The pathophysiology of paradoxical low-gradient (LG) severe aortic stenosis (SAS) remains controversial. As low transvalvular flow has been implicated, we sought to investigate the impact of left ventricular outflow tract (LVOT) ellipticity on the estimation of the LV stroke volume, the calculation of the aortic valve area (AVA) by use of the continuity equation and on AS severity grading.

**Methods:**

We studied 190 consecutive patients (mean age: 72 ± 13 years; male: 57%) with SAS (indexed AVA < 0.6 cm^2^/m^2^) and preserved LV ejection fraction, including 120 patients with severe high gradient (HG) AS and 70 with severe paradoxical LG-AS. AS severity, LV volumes and LVOT ellipticity were assessed by 2D-Doppler echocardiography and cardiac magnetic resonance (CMR).

**Results:**

The LVOT exhibited an elliptical shape on CMR images, with a shorter anterior-posterior than median-lateral diameter (2.2 ± 0.2 vs 2.8 ± 0.3 cm, *p* < 0.01). Accordingly, the LVOT area measured by planimetry was larger than by 2D-echocardiography, assuming a circular orifice (4.9 ± 0.9 cm^2^ vs 3.7 ± 0.8 cm^2^, *p* < 0.01). Inputting the elliptical LVOT area into the continuity equation resulted in a 29% increase in the indexed AVA (from 0.41 ± 0.09 cm^2^ to 0.54 ± 0.10 cm^2^). Accordingly, 30 (43%) patients with severe paradoxical LG-SAS were reclassified as having moderate AS. Similar results were obtained when considering 3D-echo for direct planimetry of the LVOT in a subset of 75 patients.

**Conclusions:**

Our results confirm that the LVOT is elliptical in shape and that taking this parameter into account in the calculation of the AVA results in reclassification of 43% of patients with severe paradoxical LG-AS into moderate AS.

## Background

Several recent retrospective studies have indicated that in elderly patients, and particularly in elderly women, severe aortic stenosis (AS) is frequently associated with lower than expected mean transvalvular gradients, even in the presence of preserved left ventricular (LV) ejection fraction (EF) [1–3]. The term severe “paradoxical low-gradient (LG)” AS was recently coined to describe this new form of severe AS [4] and to differentiate it from the well-recognized “low flow - low gradient” form seen in patients with LV dysfunction.

There is considerable debate as to the mechanisms underlying severe paradoxical LG-AS [1, 4–11]. Because its prognosis has been shown to be similar to that of moderate aortic stenosis [1, 10, 11] and because it almost systematically progresses toward severe HG-AS overtime, [10, 12–14] we have recently postulated that paradoxical LG-AS might represent a transition stage between truly moderate AS (with low mean gradients and an indexed aortic valve area (AVA) ≥ 0.6 cm^2^/m^2^) and truly severe AS (with HG and AVAi < 0.6 cm^2^/m^2^). We further hypothesized that use of the continuity equation to calculate the AVA could explain why some patients transiently pass through a stage of severe paradoxical LG-AS during their disease progression [10]. There are indeed several reasons to believe that the use of the continuity equation leads to underestimation of the AVA. First, it measures the size of the functional orifice instead of that of the anatomical orifice, like direct planimetry or the Gorlin formula. This is because, in its simplified form, it neglects the coefficient of orifice contraction, a factor that compensates for the continuous convergence of fluid streamlines beyond a narrowed orifice. Under physiological flow conditions, the degree of underestimation of the anatomical orifice by the continuity equation is expected to be around 10–15% [15]. Second, in daily clinical practice, the LV stroke volume, which is at the numerator in the continuity equation, is typically calculated by multiplying the velocity time integral of the LV outflow tract (LVOT) flow velocity by the cross-sectional area of the LVOT, the latter being calculated by measuring the anterior-posterior dimensions of the LVOT and assuming a circular orifice. Several studies have recently shown that the LVOT is rarely circular and most often exhibits an elliptical shape [16–20]. Depending on the degree of ellipticity, this is likely to result in significant underestimation of both LV stroke volume and calculated AVAs and hence in inconsistent grading of the true severity of AS.

The present study was designed to test the impact of LVOT ellipticity on the Doppler-echocardiographic estimation of LV stroke volume, AVA and AS severity grading, using cardiac magnetic resonance (CMR) as a reference standard. LVOT ellipticity was measured by direct planimetry of the aortic annulus using cardiovascular magnetic resonance (CMR). LVOT ellipcity was also measured by using transthoracic 3D-echocardiography in the subset of patient in whom 3D-volumetric datasets centered on the LVOT were available.

## Methods

### Patient population

Since January 2000, all patients with valvular heart disease referred to our Institution are enrolled into the prospective SALVARE (SAint-Luc VAlve Registry) registry. Baseline demographics, clinical features, as well as echocardiographic and CMR data are collected and stored in an electronic database. Subsequent clinical, echocardiographic and CMR data are regularly updated. From the registry database, we retrospectively selected all patients with native severe aortic stenosis who were included between May1^st^, 2005 and February 30^th^, 2015. To be selected, patients needed to display a preserved LVEF (≥50%) and to have undergone CMR as part of their initial clinical workup. Patients with more than mild aortic regurgitation or more than trivial mitral regurgitation were not considered for inclusion.

### Transthoracic echocardiography (TTE)

Echocardiographic data were obtained by use of commercially available ultrasound systems. All patients underwent a comprehensive examination, including 3-dimensional echocardiography when available. All tests were conducted by experienced sonographers. Images were analysed off-line using the XCelera software (Philips Medical Systems, Andover, MA).

For assessment of AS, multiple transducer positions were systematically used to record peak aortic jet velocities [21]. The LVOT diameter was obtained from the parasternal long-axis view in mid-systole, parallel to the valve plane and immediately adjacent to the aortic leaflet insertion into the annulus [22]. The LVOT velocity was recorded from the apical window by placing the pulsed-wave-Doppler sample volume in the outflow tract, proximal to the aortic valve. Proper positioning of the sample volume was ensured by verifying the presence of smooth spectral velocity curves associated with an aortic valve closing click. Care was taken to optimize the ultrasound beam - blood flow alignment and to avoid sampling in the transvalvular jet or the proximal flow convergence region by excluding velocity curves with spectral broadening at peak ejection. The maximal velocity across the aortic valve was measured with continuous-wave Doppler from multiple positions (apical, right parasternal, suprasternal and subxyphoidal). The highest velocity signal was used to calculate peak and mean gradients. The AVA was calculated by use of the continuity equation, assuming that the LVOT area had a circular shape. In case of atrial fibrillation, five consecutive beats were systematically averaged.

Severe AS was defined as an indexed AVA < 0.6 cm^2^/m^2^ and was further stratified into subgroups with high and paradoxically low transvalvular gradients, respectively in the presence of a mean transvalvular gradient ≥ and < 40 mmHg. Patients with severe paradoxical LG-AS were further stratified into subgroups with low flow and normal flow, respectively in the presence of an indexed stroke volume ≤ 35 or > 35 mL/m^2^ [4].

Three-dimensional echocardiographic datasets centered on the LVOT were available in a subset of 75 patients and analyzed using a QLab workstation (Philips Medical Systems, Andover, MA). After proper reorientation of the imaging plane, the true short axis of the LVOT was identified just below the aortic annulus. The LVOT area was then measured in midsystole by direct planimetry of the LVOT. Minimal and maximal diameters were also measured at the same level, to calculate the ellipticity index.

### Cardiac magnetic resonance (CMR)

**Fig. 1:**
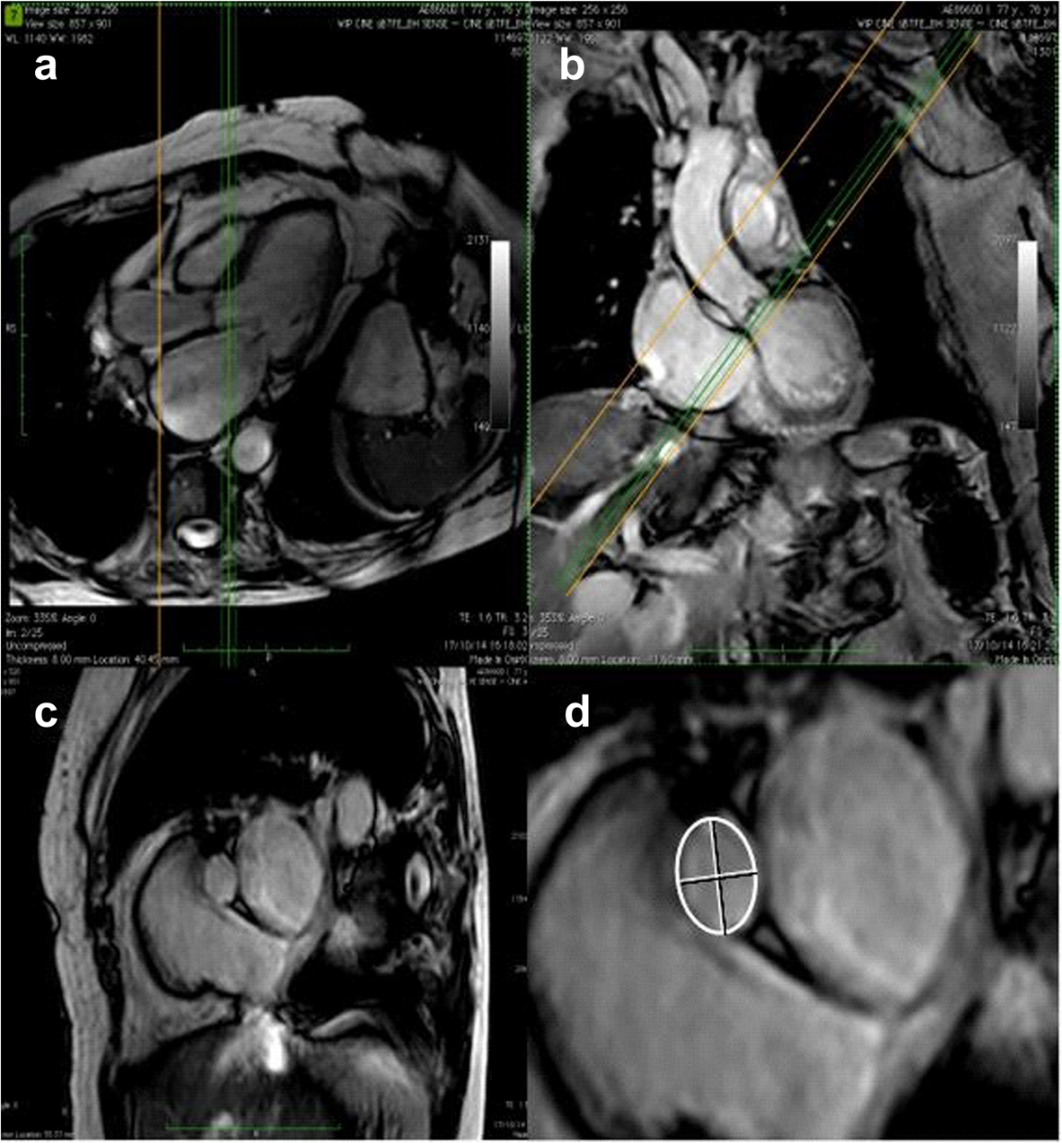
Reprentatives examples of 2 orthogonal *long-axis* (panels **a** and **b**) and the resulting *short-axis* (panels **c** and **d**) images of the LVOT by CMR illustrating its *elliptical shape*

CMR imaging was performed on a 1.5 T or 3 T magnet (Intera CV, Philips Medical Systems, Best, the Netherlands) using a five-element cardiac synergy coil for signal reception [22]. After localization of the heart using three-plane and oblique survey images, a three-chamber view and an oblique coronal view cine image of the LVOT were prescribed. These images were used as localizers to prescribe six contiguous cross-sectional cine images of the aortic valve between the LVOT and the tips of the aortic valve. The cine images were acquired using a multislice cine steady-state free precession (SSFP) pulse sequence during repeated breathholds. Slice thickness was 5 mm and slice spacing was 0 mm. Data were stored in an electronic database and analyzed off-line using commercially available software (Osirix Viewer v.5.7, Pixmeo, Bernex, Switzerland). The LVOT contours were traced manually after zooming on the area of interest (Fig. 1). LVOT minimal and maximal diameters were measured at the same level, to calculate the ellipticity index. LV volumes and ejection fraction were computed semiautomatically with manual corrections. The LV stroke volume was calculated as the difference between LV end-diastolic and end-systolic volumes. Finally, the AVA was measured by direct planimetry of the maximal opening of the aortic valve tips in systole, as previously described [23].

### Fusion of Doppler with CMR data

By combining hemodynamic echocardiographic data and LVOT area measured on CMR images, the fused indexed AVA was calculated introducing the CMR-derived LVOT area in the continuity equation.

### Statistical analysis

All analyses were performed using the SPSS v19.0 (SPSS Inc., IBM, Chicago, IL). Continuous variables were expressed as mean ± 1 SD when normally distributed and as median and range when non-normally distributed. Normality was assessed by use of the the Kolmogorov Smirnov test. Continuous variables were compared among groups using ANOVA when normally distributed or else using the Kruskall-Wallis test. Individual differences among groups were compared post-hoc using Tukey-Kramers test for normally distributed data with equal variances, the Games-Howell test for normally distributed data with unequal variances and the Mann-Whitney U tests (with Bonferroni correction for multiple comparisons) for non-normally distributed data. Categorical variables were expressed as counts and percentages. Categorical variables of patients in different groups were compared using *χ* ^2^ or the Fisher exact test. Correlation between CMR and echo data was evaluated by linear regression. Agreement between the two methods was assessed by Bland-Altman analysis. Intra- and interobserver variability for 2D-echocardiography, 3D-echocardiography and CMR data were assessed in a subgroup of 25 randomly selected patients by use of the intraclass correlation coefficient (ICC) and the Bland Altman method. All tests were two-sided and a *p* value of < 0.05 was considered indicative of a statistically significant difference.

## Results

Among the 273 subjects who met the inclusion criteria, 83 patients were subsequently excluded because of incomplete or poor quality echocardiographic images (*n* = 43) or more than mild aortic regurgitation or trivial mitral regurgitation (*n* = 40). The final study population thus consisted of 190 patients; of which 120 displayed a mean transaortic pressure gradient > 40 mmHg (HG-SAS) and 70 had a mean transaortic pressure gradient ≤ 40 mmHg (paradoxical LG-SAS). Patients with severe paradoxical LG-AS were further stratified into groups with normal (NF, *n* = 45) and low (LF, *n* = 25) transvalvular flow, using the previously described cut-off value of 35 mL/m^2^ [4].

### Baseline clinical, hemodynamic and echocardiographic characteristics

Are summarized in Tables 1 and 2. The three groups were comparable for most clinical and hemodynamic variables, with the exception of atrial fibrillation which was more prevalent in patients with severe paradoxical LF-LG-AS. From an echocardiographic point of view, patients with severe paradoxical LF-LG-AS exhibited smaller LV end-diastolic and end-systolic volume indices, lower indexed stroke volumes and smaller LVOT areas than patients from the two other subgroups. On the other hand, patients with severe paradoxical NF-LG-AS displayed larger indexed AVAs than patients from the two other subgroups. Similar results were obtained after exclusion of patients with atrial fibrillation.

**Table 1:** Baseline clinical and hemodynamic characteristics in the different subgroups

	Severe HG-SAS (*n* = 120)	Severe Paradoxical NF-LG-AS (*n* = 45)	Severe Paradoxical LF-LG-AS (*n* = 25)	*p*-value
Age, yrs	73 ± 12	74 ± 14	74 ± 14	0.44
Male gender, *n* (%)	77 (64%)	21 (47%)^*^	10 (40%)^*^	0.02
Body surface area, kg/m^2^	1.86 ± 0,19	1.81 ± 0,21	1.83 ± 0,20	0.83
Arterial hypertension, *n* (%)	84 (70%)	38 (84%)^*^	21 (88%)^*^	0.046
Diabetes, *n* (%)	20 (17%)	12 (27%)	6 (24%)	0.29
Hyperlipidemia, *n* (%)	86 (72%)	27 (60%)	24 (96%)^*,†^	0.01
Prior myocardial infarction, *n* (%)	5 (4%)	7 (16%)^*^	3 (13%)	0.04
Prior coronary revascularization, *n* (%)	15 (13%)	5 (11%)	5 (20%)	0.49
Atrial fibrillation, *n* (%)	2 (2%)	1 (2%)	5 (20%)^*,†^	<0.01

^*^ *p* < 0.05 vs HG-SAS; ^†^ *p* < 0.05 vs NF-PLG-SAS

**Table 2:** Baseline hemodynamic, echocardiographic and CMR characteristics in the different subgroups

	Severe HG-SAS (*n* = 120)	Severe Paradoxical NF-LG-AS (*n* = 45)	Severe Paradoxical LF-LG-AS (*n* = 25)	*p*-value
*Hemodynamic data*				
Heart rate, bpm	68 ± 10	69 ± 14	76 ± 16	0.17
Systolic blood pressure, mmHg	133 ± 17	141 ± 22^*^	139 ± 21	0.70
Diastolic blood pressure, mmHg	74 ± 11	76 ± 11	75 ± 12	0.88
*CMR findings*				
Indexed LV EDV, mL/m^2^	81 ± 18	73 ± 18^*^	67 ± 14^*,†^	<0.01
Indexed LV ESV, mL/m^2^	28 ± 10	26 ± 12	25 ± 8	0.14
Indexed SV, mL/m^2^	54 ± 10	49 ± 9	43 ± 10	<0.01
LV ejection fraction, %	59 ± 5	60 ± 6	57 ± 5	0.74
CMR LVOT diameter, mm	22 ± 2	21 ± 2	21 ± 2^*^	0.20
CMR LVOT area, cm^2^	5.0 ± 1.0	4.6 ± 0.9^*^	4.6 ± 0.7	0.04
CMR LVOT ellipticity index	1.28 ± 0.08	1.28 ± 0.07	1.30 ± 0.10	0.64
*2D-Doppler and Doppler findings*				
2D-LVOT diameter, mm	22 ± 2	22 ± 2	20 ± 1^*,†^	0.02
2D-echo LVOT area, cm^2^	3.8 ± 0.8	3.7 ± 0.6	3.3 ± 0.5^*^	0.02
3D-echo LVOT area, cm^2^	5.3 ± 1.0	4.9 ± 0.9	4.6 ± 0.9	0.36
LVOT VTI, cm/s	22 ± 4	22 ± 3	16 ± 3^*,†^	<0.01
2D-indexed SV, mL/m^2^	44 ± 9	42 ± 5	29 ± 4^*,†^	<0.01
Peak transaortic flow velocity, cm/s	472 ± 49	366 ± 36^*^	344 ± 42^*,†^	<0.01
Mean pressure gradient, mm Hg	56 ± 12	32 ± 6^*^	29 ± 7^*,†^	<0.01
2D-indexed AVA, cm^2^/m^2^	0.38 ± 0.08	0.49 ± 0.06^*^	0.39 ± 0.08^†^	<0.01
*Fused data*				
Fused indexed SV, mL/m^2^	57 ± 10	54 ± 8^*^	41 ± 5^*,†^	<0.01
Fused indexed AVA, cm^2^/m^2^	0.49 ± 0.09	0.62 ± 0.10^*^	0.54 ± 0.11^*,†^	<0.01

*Abbreviations*: *AVA* aortic valve area, *ERO* effective orifice area, *EDV* end-diastolic volume, *ESV* end-systolic volume, *LV* left ventricular

^*^ *p* < 0.05 vs HG-SAS; ^†^ *p* < 0.05 vs NF-PLG-SAS

### LVOT dimensions by 2D-echocardiography and CMR

**Fig. 2:**
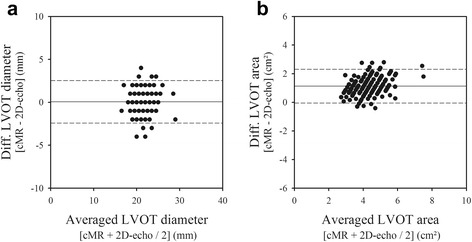
Bland-Altman *plots* comparing *2D-echo* and *CMR* measurements of the *left ventricular outflow tract* (LVOT) *anterior-posterior diameters* and *cross-sectional area*. On average, *2D-echo* underestimated the LVOT area by 29% compared with *CMR*

As shown in Fig. 1, the LVOT exhibited an elliptical shape on short-axis CMR images, with a shorter anterior-posterior than median-lateral diameter (2.2 ± 0.2 vs 2.8 ± 0.3 cm, *p* < 0.001), yielding an averaged ellipticity index of 1.28 ± 0.08. Although the anterior-posterior LVOT dimensions by 2D-echocardiography and CMR were quite similar (2.2 ± 0.2 vs 2.2 ± 0.2 cm, *p* = 0.45), with a mean bias of 0.1 ± 1.2 mm (Fig. 2a), the LVOT area measured by 2D-echocardiography, assuming a circular orifice, was significantly smaller than that measured by planimetry on CMR images (3.7 ± 0.8 vs 4.9 ± 0.9 cm^2^, *p* < 0.01) with a mean bias of 1.1 ± 0.6 cm^2^ (Fig. 2b).

### Stroke volume and continuity equation-derived AVA using 2D-echocardiography and CMR LVOT areas

**Fig. 3:**
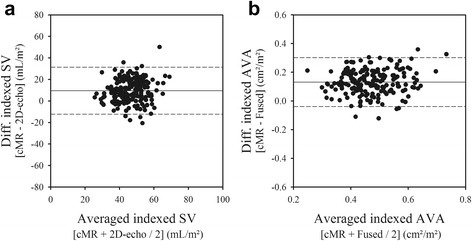
Bland-Atman *plots* comparing *2D-echo* and *CMR* measurements of indexed stroke volume (panel **a**) and of indexed *AVA* (panel **b**). On average, 2D-echo underestimated the indexed stroke volume and AVA by 29% compared with *CMR*

As shown in Fig. 3, the indexed stroke volume measured with 2D-Doppler echocardiography, assuming a circular LVOT, was significantly lower than that measured by CMR (42 ± 9 vs 51 ± 11 mLs/m^2^, *p* <0.01), with a mean bias of 10 ± 11 mLs/m^2^. Similarly, the indexed AVA calculated by use of 2D-Doppler echocardiography and the continuity equation was also smaller than the indexed AVA measured using CMR and direct planimetry of the anatomical orifice (0.41 ± 0.09 vs 0.54 ± 0.10 cm^2^/m^2^, *p* < 0.01), with a mean bias of 0.13 ± 0.09 cm^2^/m^2^.

**Fig. 4:**
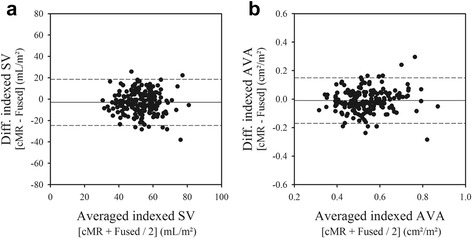
Bland-Atman *plots* comparing *fused* and *CMR* measurements of indexed stroke volume (panel **a**) and of indexed *AVA* (panel **b**). On average, inputting the planimetered LVOT area into the calculation of the indexed stroke volume by 2D-echo, corrected the underestimation of the indexed stroke volume and AVA by 2D-echo as compared to CMR

**Fig. 5:**
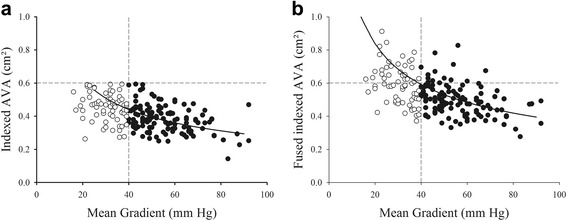
Indexed AVA (panel **a**) and fused indexed AVA (panel **b**) vs. mean gradient among 120 with HG-SAS and 70 patients with paradoxical LG-SAS. The predicted values from the fitted *curve* of the study population are presented

Inputting the CMR planimetered LVOT area into the calculation of the LV stroke volume resulted in a 29% increased of the indexed stroke volume (from 42 ± 9 to 54 ± 11 mL/m/^2^) and indexed AVA (from 0.41 ± 0.09 cm^2^ to 0.53 ± 0.11 cm^2^/m^2^, all *p* < 0.01). As a consequence, the systematic biases seen when assuming a circular LVOT almost completely disappeared (Fig. 4). This also resulted in a significant upward shift of the indexed AVA - mean gradient relationship (Fig. 5). Fitting this relationship to a non-linear function based on the formula$A V{A}_i= p/\sqrt{\varDelta P}$ allowed calculation of the indexed AVA corresponding to a mean gradient of 40 mmHg (AVA_i__40mmHg_). Using the CMR planimetered LVOT area increased the indexed AVA_40mmHg_ from 0.41 ± 0.09 to 0.59 ± 0.11 cm^2^/m^2^.

### Impact of LVOT eccentricity on AS classification

By using the fused indexed AVA, 49 patients with severe AS were reclassified as having moderate AS (indexed AVA ≥ 0.6 cm^2^/m^2^), including 19/120 HG-SAS (16%), 5/25 paradoxical LF-LG-SAS (20%) and 25/45 paradoxical NF-LG-SAS (55%). Accordingly, the concordance between Doppler-echocardiography and CMR increased from 74 to 84%, when defining severe AS as an indexed AVA < 0.6 cm^2^/m^2^, and from 84 to 93%, when defining severe AS as either an indexed AVA < 0.6 cm^2^/m^2^or a MG > 40 mmHg (*p* < 0.01).

### 3D-echo vs CMR LVOT areas

To test the ability of 3D-echocardiography to measure the true LVOT area and hence to compensate for the inherent limitations of 2D-echocardiography, we compared the planimetered LVOT areas by 3D-echo and CMR in a subset of 75 patients in who zoomed 3D-echo datasets centered on the LVOT were available. 3D-echo and CMR LVOT areas were similar (5.1 ± 1.0 vs 4.9 ± 1.0 cm^2^, *p* = 0.48) with a mean bias of 0.1 ± 0.6 cm^2^. Inputting the 3D-echo or CMR planimetered LVOT areas into the continuity equation yielded similar indexed stroke volumes (56 ± 13 vs 55 ± 11 mLs/m^2^, *p* = 0.62) and indexed AVAs (0.56 ± 0.13 vs 0.53 ± 0.11 cm^2^, *p* = 0.22). Reclassification of AS severity based on 3D-echo and CMR derived planimetered LVOT areas yielded similar results.

### Variability of measurements

LVOT planimetry measurements by CMR had an intraobserver variability (ICC) of 0.99 with a bias of 0.06 ± 0.46 cm^2^ and interobserver variability of 0.95 with a bias of −0.18 ± 0.50 cm^2^. Intra and interobserver variability for 3D-echo measurements were 0.86 and 0.94 with bias of −0.04 ± 0.048 cm^2^ and 0.03 ± 0.98 cm^2^, respectively.

## Discussion

The aim of the present study was to test the impact of LVOT ellipticity on the estimation of LV stroke volume, on the calculation of the AVA by use of continuity equation and on AS severity grading. CMR was used as the reference method for estimation of both LV stroke volume and AVA. Our results can be summarized as follows:

- In almost every patient, the LVOT exhibits an elliptical shape, with a larger transverse than anterior-posterior diameter;
- Inputting the CMR planimetered LVOT area into the continuity equation increased the indexed stroke volume and the indexed AVA by 29% and significantly reduced the bias between echocardiographic and CMR data;
- Based on the fused indexed AVAs, 55% of patients with severe paradoxical NF-LG-AS, 20% of patients with severe paradoxical LF-LG-AS and 16% of patients with severe HG-AS were reclassified as having only moderate AS. Use of the fused indexed AVA improved the concordance between Doppler-echocardiography and CMR by an average of 10%.

### Accuracy of Doppler echocardiography in measuring LV stroke volume

Accurate measurement of the LV stroke volume is of paramount importance in the calculation of the AVA by use of the continuity equation [24]. Using 2D-Doppler echocardiography, this involves proper recording of the LVOT diameter and subvalvular velocities. The LVOT diameter is usually measured in mid-systole on zoomed long-axis images. While it is usually easy to place the anterior measurement calliper onto the anterior aortic annulus, it is often more difficult to do so at the level of the posterior annulus, owing mainly to the lack of definite anatomical landmarks permitting its identification. Uncertainties regarding the exact location of the posterior aortic annulus likely explain the large variability reported in the literature for this measurement, which ranges between from 5 to 8% [24]. Since the LVOT diameter is squared for the calculation of its cross-sectional area, measurement variability is considered as one of the largest sources of error in stroke volume estimation. Yet, it is probably not the most important one. Several recent studies have indeed demonstrated that the LVOT is elliptical rather than circular in shape, leading to significant underestimation of the LVOT cross-sectional area [16–20].

The present study fully supports these previous findings. Our observations confirm that the LVOT is elliptically shaped and that its minor axis corresponds to that measured by 2D-echocardiography. Our data also indicate that the degree of underestimation of the LVOT area by 2D-echocardiography when assuming a circular orifice is as large as 29%. Since the LVOT area is used to calculate the LV stroke volume, this parameter was proportionately underestimated. Other studies have previously reported similar underestimation of stroke volume by 2D-Doppler echocardiography [25–29].

### Impact of stroke volume underestimation on the mean gradient – aortic valve area relationship

Because stroke volume is at the numerator in the continuity equation, calculated AVAs should be underestimated as well. Large degrees of underestimation of the AVA by the continuity equation in comparison of the planimetric AVA have been described before, [23] but were usually ascribed to the omission of the coefficient of orifice contraction in the simplified continuity equation. Our data indicate that the assumption of a circular LVOT is probably the main source of underestimation of both the LV stroke volume by 2D-Doppler echocardiography and the AVA by use of the continuity equation and that neglecting the coefficient of orifice contraction is probably less important.

As expected, underestimation of both the stroke volume and the AVA by conventional 2D-Doppler echocardiography had a major impact on the relationship between the AVA and the mean transaortic pressure difference. Gorlin and Gorlin were the first to systematically study this relationship [30]. Comparing hemodynamic data obtained by cardiac catheterization with surgical or autopsy findings, they elaborated a formula aimed at predicting the anatomical aortic valve area from hemodynamic measurements. Using this formula, and assuming a physiological mean transaortic flow rate of about 250 mLs/sec, a mean transvalvular gradient of 40 mmHg should correspond to an AVA of approximately 1 cm^2^. Minners et al. demonstrated that using 2D-Doppler echocardiography instead of catheterization to calculate the AVA results in a significant downward and leftward shift of the mean gradient valve area relationship, [2] so that a mean gradient of 40 mmHg does not correspond anymore to an AVA of 1 cm^2^ but rather to an AVA of 0.81 cm^2^, when using the Gorlin formula, and 0.75 cm^2^, when using the continuity equation. This 19–25% underestimation of the expected aortic valve area when using 2D-Doppler echocardiography explains why up to 40–45% of patients with a calculated AVA < 1 cm^2^ present with lower than expected mean transaortic pressure gradients, i.e. with a mean transaortic pressure difference < 40 mmHg and why, at the opposite, very few patients with an aortic valve area > 1 cm^2^ present with a mean transaortic pressure difference > 40 mmHg (<3%). The present study confirms and extends these observations. In line with previous studies, the proportion of patients presenting with lower than expected mean transaortic pressure gradient was as high as 37%. Based on the mean gradient gradient - AVA relationship, a mean transvalvular gradient of 40 mmHg was found to correspond to an indexed AVA of 0.44 cm^2^/m^2^ (AVA_40mmHg_). Correcting for LVOT ellipticity reduced the proportion of patients presenting with lower than expected mean gradients to 25% and increased the indexed AVA_40mmHg_ to 0.59 cm^2^/m^2^.

### Impact of LVOT ellipticity on AS classification

Correcting the hemodynamic equations for the LVOT ellipticity also had a profound impact on the classification of AS patients into the different AS subgroups. Indeed, as many as 25% of patients with initially severe AS were reclassified as having moderate AS, including 16% of patients with severe HG-AS, 20% of patients with severe paradoxical LF-LG-AS and 55% of patients with severe paradoxical NF-LG-AS. As a consequence, the concordance between Doppler-echocardiography and CMR improved from 74 to 84%. Similar results were recently reported by other investigators, using either multidetector computed tomography [31] or CMR [32].

Reclassification of patients with severe paradoxical NF-LG-AS into moderate AS may have important therapeutic implications since previous studies have shown that the survival of these patients was quite similar to that of patients with moderate AS. Their valves have also been shown to contain less calcium than those from patients with severe HG-AS, which further support the contention that they most probably do not exhibit truly severe AS [33, 34].

By contrast, only a minority of patients with severe paradoxical LF-LG-AS were reclassified as having moderate AS when correcting for LVOT ellipticity. Although this could indicate that they suffer from a more severe form of AS, as would be suggested by their worse outcome in some of the previously published series, [4, 8, 9] one cannot exclude the possibility that this could simply be related to their low-flow state and the inability for their LV to open up an otherwise moderately stenotic aortic valve. This hypothesis would be consistent with their relatively low calcium content as noted by several previous investigators [31, 32].

### 3D-echocardiography to assess LVOT ellipticity

Several studies have demonstrated the accuracy of 3D-echocardiography, and particularly 3D-transesophgeal echocardiography to delineate the elliptical shape of the LVOT and to measure its true cross-sectional area [35, 36]. Our study is amongst the first to demonstrate that such measurements are feasible and accurate by use of 3D transthoracic echocardiography as well. Our data further demonstrate that 3D transthoracic echo measurements of the LVOT area compare favourably with those obtained by CMR and allow alleviating the underestimation of both the LV stroke volume and the AVA by conventional 2D-Doppler echocardiography. Accordingly, 3D-echo allowed a similar proportion of patients with severe AS to be reclassified as having moderate AS as compared with CMR.

### Study limitations

Our study has several limitations that should be acknowledged. First, this is an observational study based on the a posteriori selection of patients having undergone CMR as part of their initial workup as well as on the retrospective analysis of the CMR data. Our results thus need to be confirmed in prospective studies. Second, planimetry and continuity equation-based AVAs are not identical. As discussed above, the first measures the anatomic valve area and the second measures the effective orifice area at the level of the vena contracta. In theory, they differ by a factor known as the coefficient of orifice contraction, which compensates for the continuous convergence of fluid streamlines beyond a stenotic orifice. Although fluid dynamics studies have indicated that neglecting this coefficient could lead to a 10–15% underestimation of the true anatomical orifice when using the continuity equation, our date indicate that this is certainly not the only factor to be considered and that underestimation of the LVOT area plays, at least, an equally important role. Finally, planimetry of the LVOT by 3D-echo was only available in 75 patients. The accuracy of this measurement should thus be confirmed in larger cohorts.

## Conclusions

Our results confirm that the LVOT is elliptically shaped and that failure to take this characteristic into consideration when calculating the LV stroke volume and the AVA results in a 29% underestimation of these parameters as compared to CMR. Consequently, as many as 25% of patients with severe AS are reclassified as having only moderate AS when inputting the correct LVOT area into the continuity equation. This is particularly true in patients with severe paradoxical NF-LG-AS, of whom about 2/3 are reclassified as having only moderate AS.

## Abbreviations

3D: Three-dimensional
AS: Aortic stenosis
AVA: Aortic valve area
AVAi: Indexed aortic valve area
CMR: Cardiovascular magnetic resonance
HG: High gradient
LG: Low gradient
LV: Left ventricular
LVEF: Left ventricular ejection fraction
LVOT: Left ventricular outflow tract
